# Can Sweet Maize Act as a Trap Crop for Fall Armyworm?

**DOI:** 10.3390/plants14131944

**Published:** 2025-06-25

**Authors:** Caihong Tian, Junyi Zhang, Guoping Li, Jianrong Huang, Shaoying Wu, Xinming Yin, Hongqiang Feng

**Affiliations:** 1 Henan Key Laboratory of Agricultural Pest Monitoring and Control, Key Laboratory of Integrated Crop Pests Management on Crops in Southern Region of North China, Ministry of Agriculture and Rural Affairs, International Joint Research Laboratory for Crop Protection of Henan, Biopesticide Engineering Research Center of Henan Province, Agricultural Microbiology Innovation Center of Henan Province, No. 0 Entomological Radar Field Scientific Observation and Research Station of Henan Province, Institute of Plant Protection, Henan Academy of Agricultural Sciences, Zhengzhou 450002, China; caihongtian@hnagri.org.cn (C.T.);; 2 Sanya Nanfan Research Institute, Hainan University, Sanya 572025, China; 3 College of Food and Biological Engineering, Henan University of Animal Husbandry and Economy, Zhengzhou 450046, China

**Keywords:** *Spodoptera frugiperda*, sweet and normal maize, adult rhythm, circadian clock genes, RT-qPCR

## Abstract

Among various plants, corn is the primary host damaged by *Spodoptera frugiperda* J. E. Smith (Lepidoptera: Noctuidae). After long-term regional colonization, its larvae feed on sweet waxy corn and fresh corn for extended periods. A question arises: Does long-term feeding on different corn varieties affect their rhythms? Currently, there are no reports addressing these issues. To facilitate the formulation of effective prevention and control measures, Zhengdan 958 and Zhenghuangnuo were selected as representative varieties of normal and sweet waxy corn, respectively, for laboratory experiments. *S. frugiperda* were fed the leaves of these two corn types over nine consecutive generations, thereby establishing distinct *S. frugiperda* strains associated with each corn variety. Additionally, a strain fed an artificial diet served as the control group. Through a comparative analysis of the emergence, movement, nutritional foraging, dormancy, mating, and oviposition behaviors of adult fall armyworms from different populations, differences in the six behavioral peak times among the strains were identified. RT-qPCR analysis indicated significant differences in the expression levels of four circadian clock genes across different populations and tissues of the fall armyworm. Feeding on different host plants influenced the expression of circadian clock genes and their associated behavioral rhythms. Our study showed that sweet corn is more conducive to pupation, mating, and oviposition. Because of these differences in adult insect rhythms, sweet corn may have an impact on the reproduction of fall armyworms in the Huang–Huai–Hai corn-planting region.

## 1. Introduction

Throughout the extensive evolutionary journey of insects, the cycles of day and night, seasonal variations, and other terrestrial changes have led to the development of rhythmic behaviors, commonly referred to as biological clocks [1,2]. In moths, the primary rhythmic activities during developmental stages include pupation, eclosion, mating, and oviposition. Additionally, the behavioral rhythms exhibited by different insect species vary significantly. Even within the same species, the consistency of behavioral rhythms is influenced by numerous external factors. These variations in insect behavioral rhythms reflect their adaptations to diverse environments and contribute to the extensive variety within moth species [3,4].

The fall armyworm (FAW), *Spodoptera frugiperda* (J.E. Smith) (Lepidoptera: Noctuidae), is a significant migratory pest that affects crops globally and poses a considerable risk to food security in China [5]. Current research on the biology and rhythmic patterns of FAW primarily investigates mating, oviposition, hatching, adult lifespan, and how various feeding conditions impact its growth and development [6,7,8,9]. Significant advancements have also been made in understanding the interactions between FAW and its host plants. Studies have demonstrated that the selectivity and fitness of FAW in relation to different hosts, along with oviposition preferences and the influence of feeding on various host plants, considerably affect its growth and development [10,11,12,13,14].

Although FAW has an extensive range of hosts in the Huang–Huai–Hai region of China, it predominantly inflicts damage on maize. Trap cropping is a behavioral management strategy where a preferred host plant attracts pests away from the main crop; this method utilizes the host-finding behavior of insects to decrease pest pressure [15]. *S. frugiperda* feeding on different host plants may experience alterations in their circadian rhythms, and even minor variations in these rhythms can influence gene expression among individuals and impact a range of reproductive behaviors [16,17]. Studies have shown that geographical and host differences can affect the growth, development, and reproduction of *S. frugiperda* [18,19]. Understanding how these changes might influence the FAW’s ovarian development and subsequent reproductive success is crucial for developing effective control strategies. In this study, we aimed to investigate the ovarian development of FAW raised on different host plants and assess any potential variations in their circadian rhythms that could impact their reproductive behaviors. This study investigates sweet maize’s potential as a trap crop for *S. frugiperda* under agroecosystem conditions of the summer maize production area in the Huang–Huai–Hai region of China, which will provide valuable insights into the biology of FAW and contribute to the development of more targeted and sustainable pest management practices.

## 2. Materials and Methods

### 2.1. Insects

The initial insects of *S. frugiperda* comprised 3 to 5 instar larvae collected from maize fields in Minggang Town, Xinyang, Henan Province, in June 2019. These larvae were reared continuously over 3 generations using an artificial diet in a climate-controlled environment (PQX-280A-3H artificial climate box, Ningbo Laifu Technology Co., Ltd., Ningbo, China) maintained at a temperature of (27 ± 1) °C, with a photoperiod of 14 h of light and 10 h of dark, and a relative humidity of (75 ± 5)%. Additionally, two maize strains were reared for 9 generations on fresh corn leaves. Following pupation of the various larval strains, male and female pupae were separated into different containers, while the control group consisted of adults of *S. frugiperda* raised on an artificial diet in the laboratory.

### 2.2. Observation of Adult Behavior and Rhythm

The pupae of *S. frugiperda* were categorized by gender, and those ready for eclosion were chosen and placed in 50 mL insect boxes (dimensions: bottom × upper bottom × height = 28 mm × 38 mm × 30 mm) [20]. Each box contained two pupae, which were organized neatly, and an infrared camera was used for video recording, with observations made every hour.

The eclosion of the fall armyworm was conducted with a pairing ratio of female to male moths of 1:1, referenced by *Helicoverpa armigera* Hübner density protocols to minimize crowding effects and behavioral artifacts [21]. Transparent packaging boxes measuring 10 cm on each side were used, with one pair per box. A total of 11 treatments were set up, each repeated three times, resulting in 33 pairs. To provide nutrition for the adults, a cotton ball soaked in 10% sucrose water was placed inside each box, adult behavior was observed and recorded every 20 min using the infrared camera, and observers were blinded during behavioral recording.

### 2.3. Differentiation of Behavioral Characteristics

The activities of adult *S. frugiperda* were categorized into five distinct types. 1. Movement: Adults either crawl or fly within the insect box, with activity lasting over 3 s. 2. Nutritional Supplementation: Adults feed on sucrose water placed on defatted cotton. 3. Resting: Adults remain stationary in the insect box (excluding feeding, mating, and oviposition). 4. Mating: The females and males align their abdomens closely, forming a V-shape or type I configuration. 5. Oviposition: The tip of the adult’s abdomen moves as it lays eggs. Additionally, the eclosion and behavioral patterns of *S. frugiperda* reared for one generation on corn leaves were observed and documented, and these were compared to those of a strain reared for nine generations on the same diet.

### 2.4. Determination of Ovarian Development

Pupae that exhibited a normal appearance and consistent size were chosen and placed into a dipping box. Following eclosion, no nutritional supplementation was provided. At 8:30 a.m. on the second day post-eclosion, females were randomly selected for dissection to assess ovarian development. During dissection, the wings of the female moths were first removed, and scales were collected in a wax plate, secured with an insect needle. Using tweezers, the membrane from the 8th internode of the tail was carefully torn to the midline of the dorsal side, and the body wall was anchored on both sides using an insect needle. The ovary was extracted with a hook needle and weighed on an analytical balance (Mettler Toledo ME204T), recorded as M0. It was then placed in a wax plate containing 5% pentanediol for observation and photography with a super-depth-of-field microscope (Moticam Pro S5 Lite). The ovarian duct’s length was measured using Image J 1.52a (https://javadoc.io/doc/net.imagej/ij/1.52a, 19 June 2025) after dissection and imaging under a binocular stereomicroscope (Shimadzu Kalnew Stereo Microscope (20X/40X)). The fat body was removed and placed into a fresh culture dish. Excess water and the free fat body were absorbed with filter paper and weighed, denoted as M1. The weight of the fat body was determined by calculating the following difference: M0–M1. Ten individuals were dissected for each treatment, with three repetitions conducted.

### 2.5. In Vitro Expression of Rhythm-Related Genes

The male and female adults of *S. frugiperda* after 3 days of eclosion were taken, and the RNA of different parts was extracted at 8:00 a.m. to prepare cDNA libraries. For generating the first-strand cDNA, 2 µg of RNA of female and male *S. frugiperda* adults was reverse-transcribed in a 20 μL reaction volume using the PrimeScript™ RT reagent Kit with gDNA Eraser (Perfect Real Time). To confirm the single-amplicon product for qRT-PCR and the bio-specificity for *S. frugiperda*, gradient PCR was performed for all primer pairs and optimized for a 60 °C annealing temperature for all primer pairs. PCR amplicon products were run on 1.5% 1xTAE agarose gel electrophoresis and Sanger-sequenced (Biological Engineering (Shanghai) Co., Ltd., Shanghai, China).

qRT-PCR was performed using the SYBR^®^ Premix Ex Taq™ II (Tli RNaseH Plus) (TaKaRa) according to the manufacturer’s protocol, in the StepOne Plus Real-time PCR system (Applied Biosystems, Waltham, MA, USA), with the following PCR-cycling conditions: 95 °C for 10 s, 40 cycles of 95 °C for 30 s, and 60 °C for 30 s with a concentration of 200 nM of each primer pair. The mean threshold cycle (Ct) was measured by three replicates of each gene to test sample contamination and dimer formation, and nuclease-free water as the template was included in all qRT-PCR experiments as the negative control. The primer design of four circadian clock genes like Period (Per), Cryptochrome (Cry), Cycle (Cyc), and Clock (Clk) in *S. frugiperda* was established [22]. The 2-ΔΔ Ct method [23] was used to calculate the relative expression levels of individual genes. The actin gene (unigene 62750) was chosen as the endogenous reference gene in the qRT-PCR experiment as previously demonstrated. All primers pairs for qRT-PCR are listed in Table 1.

### 2.6. Data Analysis

The experimental data were statistically analyzed using SPSS 22.0. One-way analysis of variance was used. The difference between the same gender was tested by Tukey’s method. The significant difference between male and female was tested using the *t*-test.

## 3. Results

### 3.1. The Effect of Different Maize Cultivars on the Emergence Rhythm of the First Generation of the S. frugiperda Strain

The emergence times of various *S. frugiperda* strains varied, yet a distinct peak was observed. For the first generation of the Zhengdan 958 strain and the artificial diet feed strain, the emergence peak occurred between 0:00 and 1:00, while the first generation of the Zhenghuangnuo strain peaked from 23:00 to 0:00. The emergence peak of the Zhenghuangnuo strain occurred earlier than that of the grain corn strain. All three strains exhibited their emergence peaks during the dark period.

**Figure 1:**
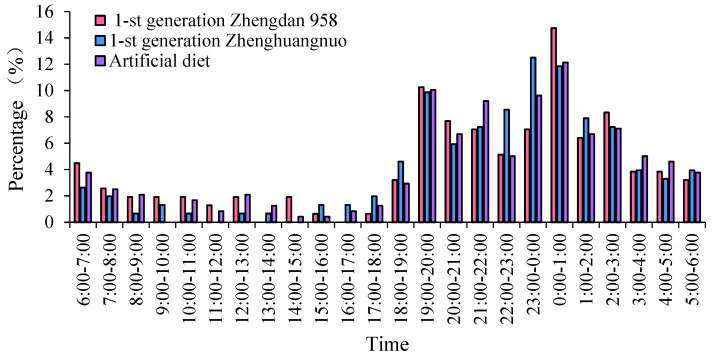
The adult emergence rhythm of the first-generation population of *S. frugiperda.* Data in the figure are mean ± SE. Different capital and lowercase letters above the bars indicate extremely significant (*p* < 0.01) and significant differences (*p* < 0.05) in the different strains of *S. frugiperda* adults (ANOVA and Duncan’s new multiple range test), respectively.

The emergence duration for each strain was further recorded. The Zhengdan 958 strain and the artificial diet feed strain exhibited emergence periods of approximately two hours, centered around their respective peaks. The Zhenghuangnuo strain, on the other hand, displayed a slightly longer emergence period, spanning nearly three hours around its peak. The grain corn strain showed the most variable emergence times, with individuals emerging over a broader window of several hours, primarily during the dark period. The synchronization of emergence within strains, especially during the dark phase, suggests the potential for a reduced predation risk and increased mating opportunities (Figure 1).

### 3.2. The Impact of Different Maize Cultivars on the Activity Patterns of FAW Adults in the First-Generation Strain

**Figure 2:**
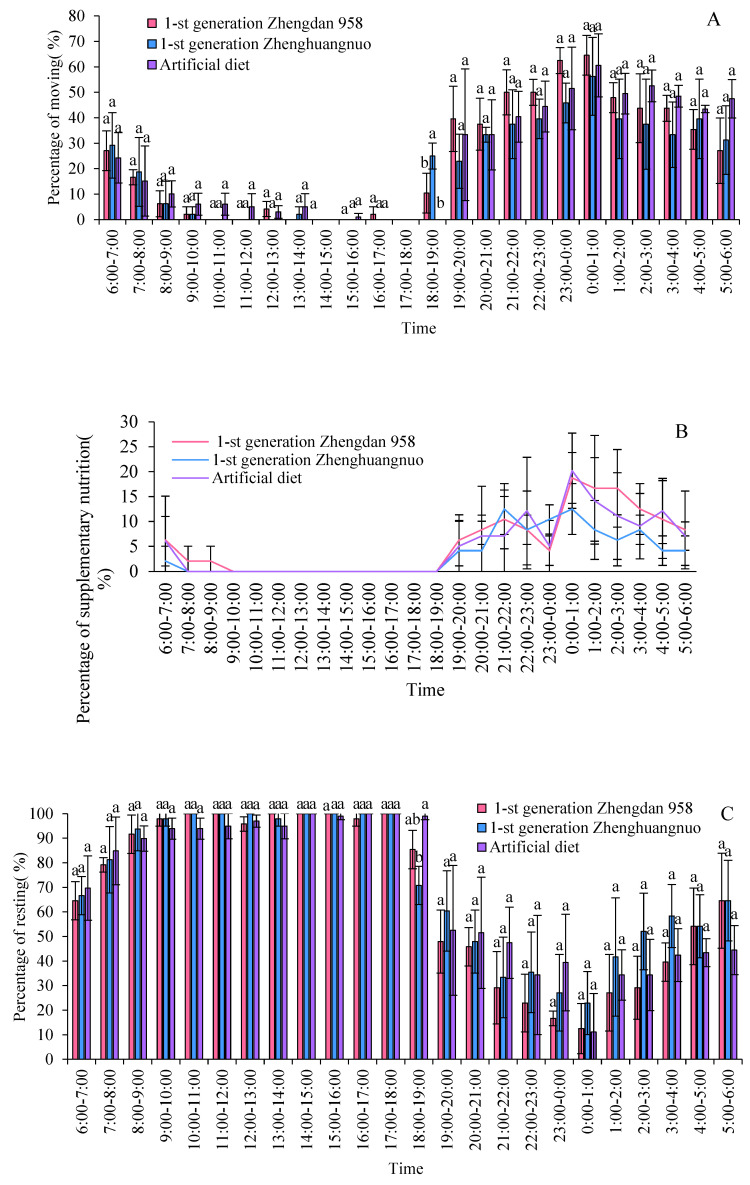

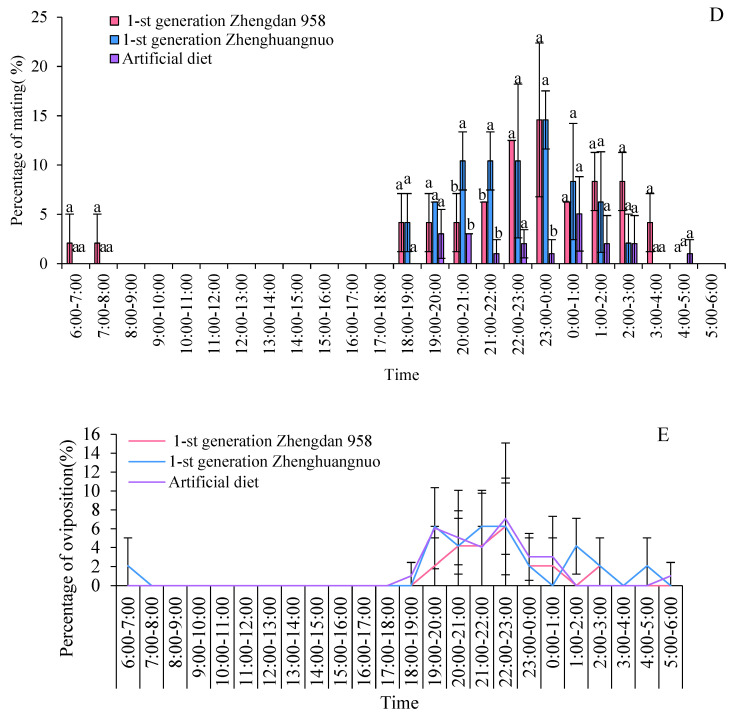
Rhythms of emergence, movement, resting, mating, and oviposition for different strains of *S. frugiperda* adults in the first generation. (**A**–**C**) Emergence, movement, and resting rhythms of the first-generation *S. frugiperda* strains, respectively. (**D**,**E**) Mating and oviposition rhythms of different *S. frugiperda* strains in the first generation, respectively. The different lowercase letters above the column represent the significance of differences between strains.

During the light–dark cycle, different strains of the fall armyworm exhibit distinct activity patterns, accompanied by significant periods of rest. The activity levels of all three strains significantly decrease after 8:00. However, the activity of the first generation of Zhengdan 958 and Zhenghuangnuo strains significantly increases at 18:00 in the evening, while the artificially fed strain reaches its peak activity at 19:00. During the dark period, each strain shows a pattern of gradually increasing activity followed by a decline, with the artificially fed strain exhibiting a more gradual decrease. Among all strains, the highest frequency of activity occurs between 00:00 and 01:00. Before the commencement of the dark period, the time at which adult activity begins to rise varies among the three strains: the Zhengdan 958 and Zhenghuangnuo strains begin to rise 2 h before the dark period, while the artificially fed strain rises 1 h before. The resting behavior of all the adult strains is inversely proportional to the amount of activity, with higher frequencies during the light period and lower frequencies during the dark period (Figure 2C).

Supplementary nutritional behavior among adult *S. frugiperda* species primarily occurred in darkness, with minimal activity during the light hours. The peak for supplementary feeding was between 00:00 and 01:00 for both the first generation of the Zhengdan 958 strain and the artificial diet feed strain, whereas the Zhenghuangnuo strain peaked at 21:00 to 22:00 and again at 00:00 to 01:00.

Additionally, the frequency of supplementary feeding was notably higher in the Zhengdan 958 strain compared to the other two strains, suggesting potential differences in nutritional requirements or feeding habits among these variants. For all strains, the amount of food consumed during the supplementary feeding periods was consistent across replicates, indicating a reliable and reproducible feeding pattern.

All three strains exhibited a distinct peak in mating behavior: the Zhengdan 958 and Zhenghuangnuo strains peaked between 23:00 and 00:00, while the artificial diet strain peaked between 00:00 and 01:00. Spawning peaks were observed as follows: the first-generation Zhengdan 958 strain had one peak (22:00 to 23:00), the first-generation Zhenghuangnuo strain had two peaks (21:00 to 22:00 and 22:00 to 23:00), and the artificial diet strain also had two peaks (19:00 to 20:00 and 22:00 to 23:00). The highest spawning frequency for each strain was recorded between 22:00 and 23:00 (Figure 2D).

In addition, the ovarian development of the insects was monitored, revealing that all strains reached the maximum ovarian maturity during their respective spawning peaks. The ovarian development index for each strain correlated positively with their spawning frequencies, indicating a strong relationship between ovarian maturity and reproductive activity.

### 3.3. Impact of Various Maize Cultivars on the Mating Duration of Adult S. frugiperda from a First-Generation Strain

**Figure 3:**
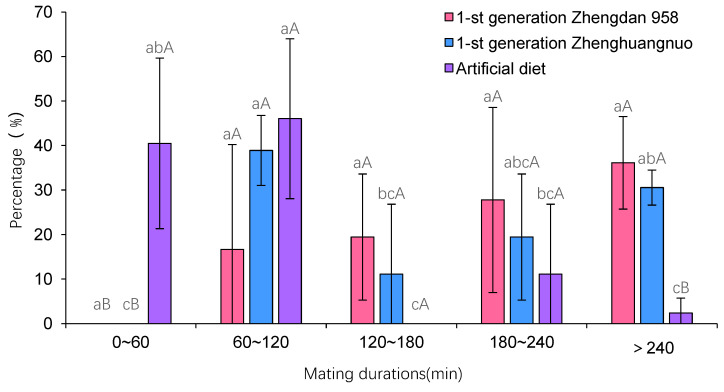
The mating duration of *S. frugiperda* adults in the first-generation population. Data in the figure are mean ± SE. Different lowercase and capital letters above the bars indicate significant differences (*p* < 0.05) and extreme significant (*p* < 0.01) in the different strains of *S. frugiperda* adults respectively (ANOVA and Duncan’s new multiple range test).

The mating duration for *S. frugiperda* across the three strains was at least 60 min. Specifically, the first generation of Zhengdan 958 averaged 206.18 ± 90.43 min, the first generation of Zhenghuangnuo averaged 189.90 ± 116.23 min, and the artificial diet strain averaged 83.35 ± 52.32 min. The mating times for both the first-generation Zhengdan 958 and Zhenghuangnuo strains were significantly longer than that of the artificial diet strain (*p* = 0.002) (Figure 3).

Across the three strains, the Zhengdan 958 strain exhibited an average copulation frequency of 1.67 ± 0.71 times per female, while the Zhenghuangnuo strain averaged 1.58 ± 0.65 times per female. The artificial diet strain showed a slightly lower frequency at 1.42 ± 0.50 times per female. However, no statistically significant differences were observed among the strains in terms of copulation frequency (*p* = 0.347) (Figure 3).

### 3.4. The Impact of Various Maize Cultivars on the Expression of Rhythm Genes in the First Generation of S. frugiperda Adults

The expression levels of the four circadian clock genes varied among different strains. In comparison to the females of the artificial diet strain, *per* gene expression was reduced in the antennae, head, flight muscle, and ovary of the first generation of the Zhengdan 958 strain, while it increased in the leg. For the first generation of the Zhenghuangnuo strain, *per* gene expression decreased in the antennae, head, and flight muscle but rose in the leg and ovary. *clk* gene expression was lower in the antennae, leg, and ovary of the first generation of the Zhengdan 958 strain, but higher in the head and flight muscle. In the first generation of the Zhenghuangnuo strain, *clk* expression decreased in the antennae and ovary, while it increased in the head, leg, and flight muscle. *cry* gene expression diminished in the antennae, leg, and flight muscle of the first generation of the Zhengdan 958 strain, while it increased in the head and ovary. Similarly, in the first generation of the Zhenghuangnuo strain, *cry* gene expression decreased in the antennae and leg, but increased in the head, flight muscle, and ovary. *cry* gene expression rose in the antennae, head, and ovary of the first generation of the Zhengdan 958 strain and also increased in the head and ovary. In contrast, the expression level of *cry* in the antennae and ovaries of the first generation of the Zhenghuangnuo strain increased, while it decreased in the head, leg, and flight muscle.

In comparison to the male of the artificial diet strain, the expression of the *per* gene was reduced in the antenna and head of the first generation of the Zhengdan 958 strain, while it was elevated in the leg, flight muscle, and testis. Conversely, the expression level rose across all regions in the first generation of the Zhenghuangnuo strain. For the *clk* gene, expression decreased in the antennae of the first-generation Zhengdan 958 strain but increased in the head, leg, flight muscle, and testis. In the first generation of the Zhenghuangnuo strain, the expression of *clk* reduced in both the antennae and testis but was heightened in the head, leg, and flight muscle. The *cry* gene exhibited an increase in expression throughout all parts of the first-generation Zhengdan 958 strain. Similarly, in the first generation of the Zhenghuangnuo strain, *cry* expression rose in the antennae, leg, flight muscle, and testis, while it dropped in the head. The *cyc* gene’s expression increased in the antenna and head of the first-generation Zhengdan 958 strain but decreased in the leg, flight muscle, and testis. In contrast, the expression of *cyc* was heightened in the antennae of the first-generation Zhenghuangnuo strain, yet it was reduced in the head, leg, flight muscle, and testis.

**Figure 4:**
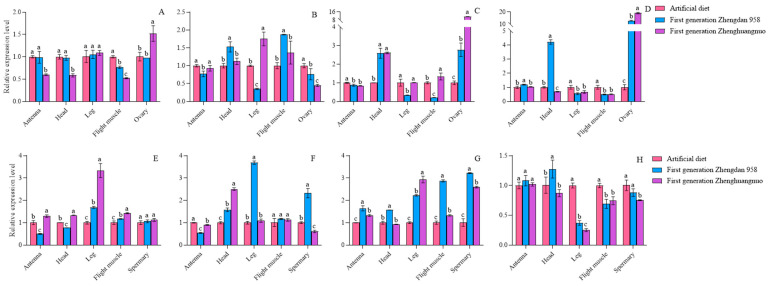
The relative levels of *circadian clock* gene expression in adult individuals of the first generation of the *S. frugiperda* strain. Data in the figure are mean ± SE. Different lowercase letters above the bars indicate significant differences (*p* < 0.05) in the different strains of *S. frugiperda* adults (ANOVA and Duncan’s new multiple range test), respectively. The expression levels of Per, Clk, Cry, and Cyc in adult females are labeled (**A**–**D**), whereas in adult males, they are indicated by (**E**–**H**).

When comparing the artificial diet strain to the first generation of both the Zhengdan 958 and Zhenghuangnuo strains, variations in circadian gene expression levels were observed, with significant differences in certain genes across the strains. Notably, *cry* gene expression in the ovary of the first-generation Zhenghuangnuo strain was found to be 11.39 times greater than that of the artificial diet strain. In the ovaries of the first-generation Zhengdan 958 and Zhenghuangnuo strains, *cyc* gene expression was 12.94 and 19.08 times higher, respectively, than in the artificial diet strain, although most expression levels fluctuated between 0.5 and 2 times (Figure 4).

### 3.5. Impact of Various Maize Cultivars on Emergence Timing of S. frugiperda

**Figure 5:**
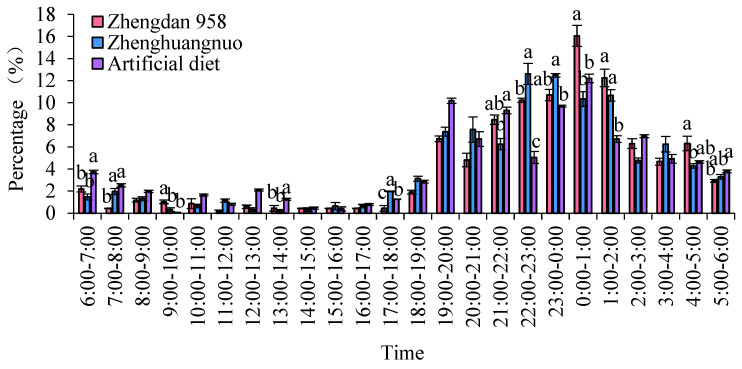
The adult emergence pattern of *S. frugiperda.* Data in the figure are mean ± SE. Different lowercase letters above the bars indicate significant differences (*p* < 0.05) in the different strains of *S. frugiperda* adults (ANOVA and Duncan’s new multiple range test).

Different strains of *S. frugiperda* exhibited varying emergence times, although a clear peak was noted. The emergence peaks for the Zhengdan 958 strain, Zhenghuangnuo strain, and artificial diet feed strain occurred between 0:00 and 1:00, 22:00 and 23:00, and 0:00 and 1:00, respectively. All three strains peaked during the dark period, but the Zhengdan 958 and Zhenghuangnuo strains showed a more concentrated emergence, while the artificial diet strain had a more scattered emergence. Notably, the emergence peak of the Zhenghuangnuo strain was 2 h earlier than that of both the Zhengdan 958 strain and the artificial diet strain (Figure 5).

### 3.6. Eclosion Patterns of Male and Female Adults Across Different Strains

**Figure 6:**
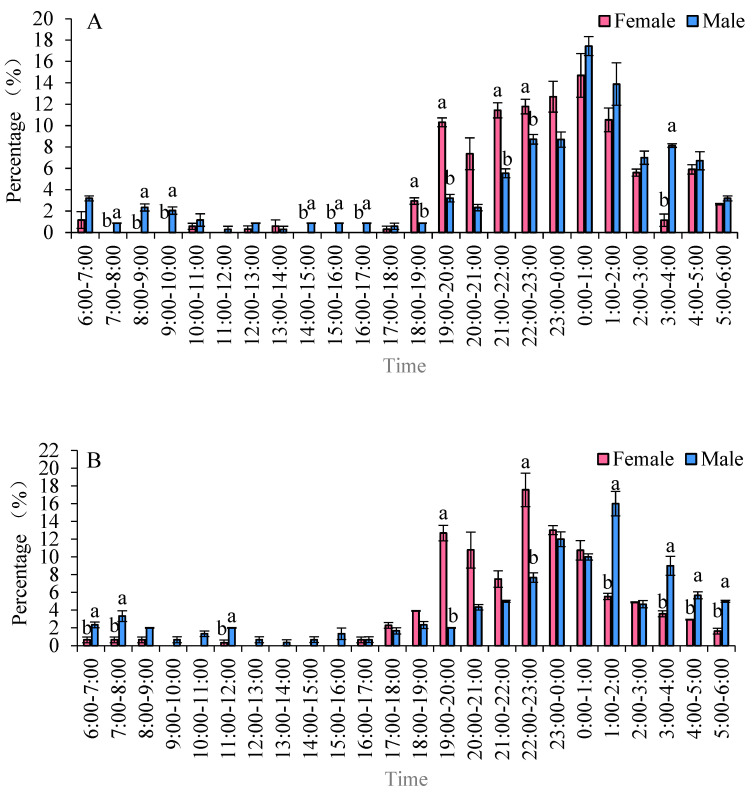

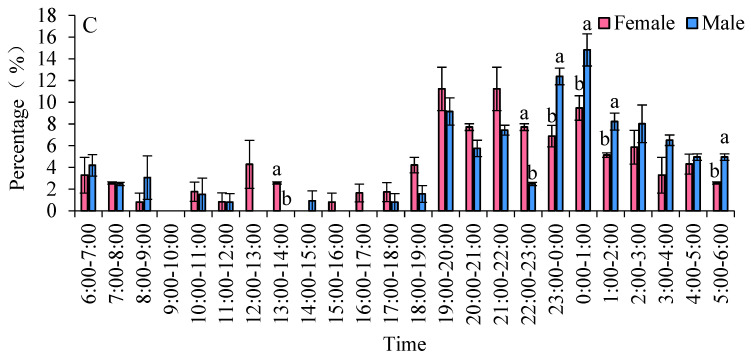
The emergence patterns of adult *S. frugiperda* across various strains. (**A**) Zhengdan 958 strain; (**B**) Zhenghuangnuo strain; (**C**) artificial diet feed strain. Data in the figure are mean ± SE. Lowercase letters above the bars indicate significant differences (*p* < 0.05) in the male and female adults (ANOVA and Duncan’s new multiple range test).

The eclosion peaks of *S. frugiperda* varied among different strains, with females emerging earlier than males. In two maize strains, the eclosion of females occurred before that of males. Specifically, in the Zhengdan 958 strain, the eclosion peaks for males and females occurred between 18:00 and 19:00 and from 00:00 to 01:00, respectively. During the time frame of 18:00 to 01:00, the eclosion rates were 81.26% for males and 46.80% for females. For the Zhenghuangnuo strain, the peak eclosion times were 19:00 to 20:00 and 22:00 to 23:00 for males, followed by 23:00 to 00:00 and 01:00 to 02:00 for females. The eclosion percentages between 19:00 and 23:00 were 48.53% for males and 18.87% for females. In the artificial diet strain, the peaks occurred from 19:00 to 20:00 and from 21:00 to 22:00 for males, with females emerging from 19:00 to 20:00 and 00:00 to 01:00. The eclosion rates during 19:00 to 22:00 were 29.91% for males and 22.13% for female adults (Figure 6).

### 3.7. Impact of Various Maize Cultivars on Circadian Activity Patterns of S. frugiperda Adults

Different strains of *S. frugiperda* exhibited a resting phase during light periods and an active phase during dark periods. The activity levels of all three strains significantly declined, starting at 8:00, and rose notably after 19:00. However, the Zhengdan 958 strain displayed a pronounced peak of activity between 7:00 and 8:00, marking the highest activity time of the day. The Zhenghuangnuo strain’s activity frequency initially increased slowly before stabilizing during the dark period. In contrast, the artificial diet feed strain’s activity frequency showed a gradual rise followed by a decrease in the dark period, peaking between 0:00 and 1:00. All three strains began to show increased activity before the onset of darkness, although the timings varied: Zhengdan 958 at 2 h, Zhenghuangnuo at 3 h, and the artificial diet strain at 1 h before the lights went out. The resting behavior of the adults in these strains was inversely related to their activity patterns, revealing higher and lower resting frequencies during light and dark periods, respectively.

Supplementary feeding behaviors in adult *S. frugiperda* predominantly occurred during dark periods, with negligible activity in light periods. The peaks for supplementary feeding were from 3:00 to 5:00 for the Zhengdan 958 strain, from 3:00 to 4:00 for the Zhenghuangnuo strain, and from 0:00 to 1:00 for the artificial diet feed strain.

Both the Zhengdan 958 and Zhenghuangnuo strains exhibited two mating peaks, whereas the artificial diet feed strain showed a single noticeable peak. The mating peaks for the Zhengdan 958 strain occurred between 20:00 and 21:00 and between 2:00 and 3:00, accounting for 10.10 and 6.06% of total mating events, respectively. The Zhenghuangnuo strain peaked at 18:00–19:00 and 2:00–3:00, representing 5.05 and 9.09%, respectively. The artificial diet feed strain’s mating peak was from 0:00 to 1:00, making up 5.05%. The mating patterns of the strains reared on maize were more concentrated, while those of the artificial diet feed strain were more dispersed.

**Figure 7:**
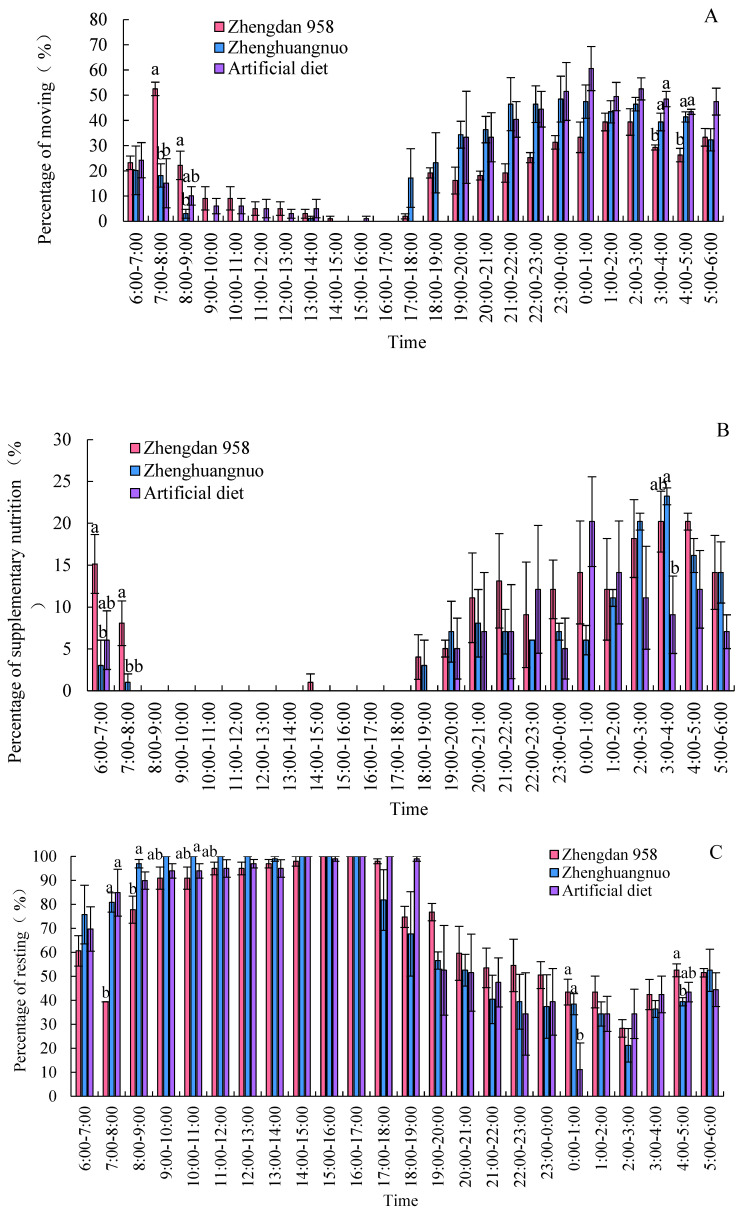

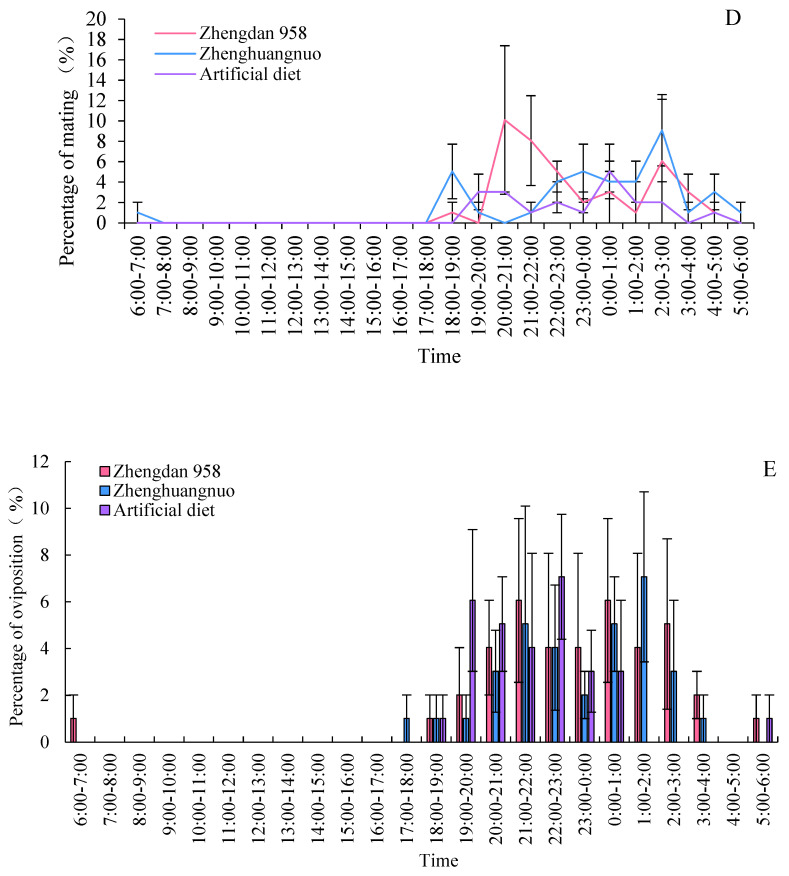
Behavior rhythm of *S. frugiperda* adults. (**A**–**C**) Emergence, movement, and resting rhythms of *S. frugiperda* strains, respectively. (**D**,**E**) Mating and oviposition rhythms of different *S. frugiperda* strains, respectively. Data in figure are mean ± SE. Different lowercase letters above bars indicate significant differences (*p* < 0.05) in the different strains of *S. frugiperda* adults (ANOVA and Duncan’s new multiple range test).

In the Zhengdan 958, Zhenghuangnuo, and artificial diet feed strains, there were three (from 21:00 to 22:00, 00:00 to 01:00, and 02:00 to 03:00), two (from 21:00 to 22:00 and 01:00 to 02:00), and two spawning peaks (from 19:00 to 20:00 and 22:00 to 23:00), respectively. The spawning peaks for the two maize strains occurred approximately two hours later than those of the artificial diet feed strain (Figure 7).

### 3.8. Effects of Different Maize Cultivars on Mating Duration of Adult S. frugiperda

**Figure 8:**
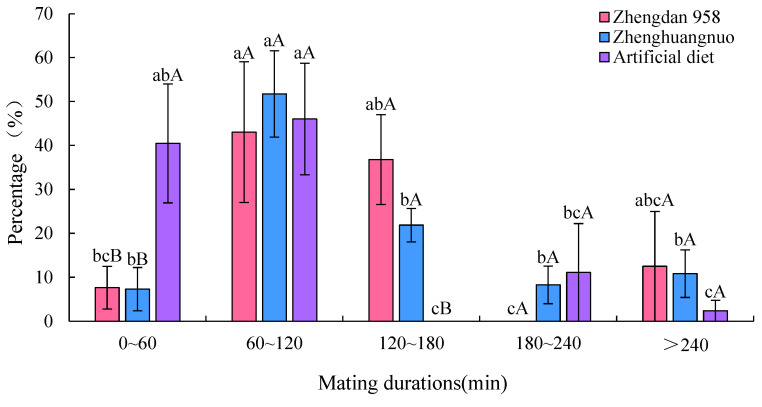
Mating duration times of *S. frugiperda* adults. The lowercase letters in the diagram indicate the significance of differences in mating time within the same strain, while the uppercase letters denote the significance of differences in the percentage of mating time among various strains during the same interval.

The average mating duration for *S. frugiperda* across the three strains was at least 60 min. Specifically, the average mating times were 143.73 ± 15.53, 1286.45 ± 20.66, and 70.87 ± 2.45 min for the Zhengdan 958, Zhenghuangnuo, and artificial diet strains, respectively. The mating duration for the Zhenghuangnuo strain was significantly longer than those of the Zhengdan 958 and artificial diet strains (*p* = 0.02) (Figure 8).

### 3.9. Daily Time Allocation of Adult Behavior of S. frugiperda

**Figure 9:**
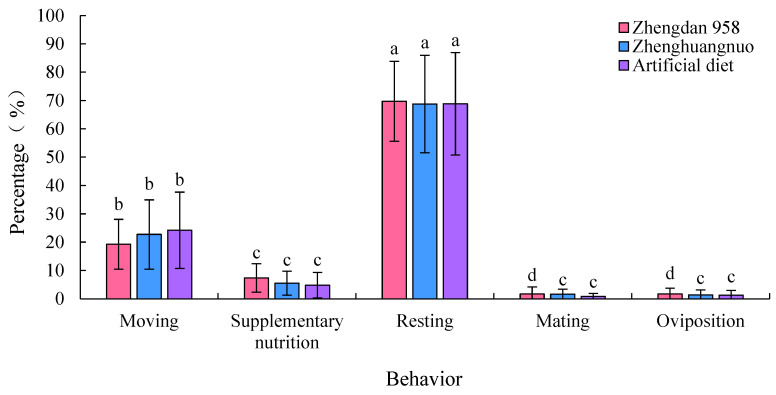
The time allocation of behavior of *S. frugiperda* adults. The lowercase letters above the bars indicate significant differences in the proportion of time allocation of each behavior among the same strain.

The adult *S. frugiperda* exhibited the highest proportion of resting behavior among the various strains, which was significantly greater than that of other behaviors (*p* < 0.05). This was followed by moving behavior, which was also significantly more prevalent than supplementary nutrition, mating, and oviposition behaviors (*p* < 0.05). Specifically, the resting behavior proportions for the Zhengdan 958, Zhenghuangnuo, and artificial diet strains were 69.74, 68.77, and 68.86%, respectively. The three strains showed limited mating and oviposition behaviors, and there were no significant differences in the distribution of various behaviors across the strains (Figure 9).

### 3.10. Effects of Different Maize Cultivars on Ovarian Development of S. frugiperda

**Figure 10:**
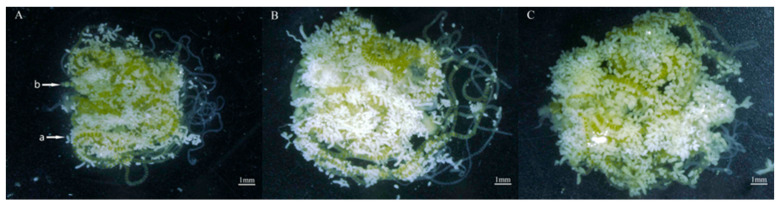
Ovaries of *S. frugiperda* reared on two types of corn leaves and artificial diet. (**A**) Zhengdan 958 strain; (**B**) Zhenghuangnuo strain; (**C**) artificial diet feed strain. a: fat body; b: eggs. Bar = 1 mm.

After nine generations of continuous feeding on two different cultivars of corn, the newly emerged female moths were examined through ovarian dissection. The ovary weights were 13.80, 22.10, and 17.40; the fat body weights were 12.20, 13.80, and 6.7; the fat body contents % were 35.22, 43.36, and 31.54%; and the ovarian tube lengths were 39.95, 41.98, and 35.66 for Zhengdan 958, Zhenghuangnuo, and the artificial diet, respectively. The results indicated that the corn cultivars did not significantly influence the ovarian development stage of *S. frugiperda*. When fed corn leaves and an artificial diet, both strains of *S. frugiperda* displayed an ovarian development grade of I (the transparent milky stage). However, the strain of sweet corn exhibited a superior ovarian tube length compared with the other two strains. Conversely, the fat body weight and fat body content of the sweet and normal corn strains were significantly heavier than those of the artificial diet strain (*p* < 0.05) (Table 2, Figure 10).

### 3.11. Effects of Different Maize Cultivars on the Expression of Rhythm Genes in Different Tissues of S. frugiperda Adults In Vitro, as Studied by RT-qPCR

The expression levels of four circadian clock genes in various strains and tissues of *S. frugiperda* were examined using RT-qPCR. Significant differences were observed in the expression of *per*, *clk*, *cry*, and *cyc* genes across different strains and tissues. In the artificial diet strain, the *per* gene exhibited the highest expression in flight muscle, followed by the head and antennae. The *clk* and *cry* genes showed their highest expression in the antennae, while the *cyc* gene was predominantly expressed in the flight muscle. For male insects, the *per* gene had peak expression in the head, with the *clk* gene also highest in the antennae. The *cry* and *cyc* genes were most highly expressed in the antennae, followed by the head.

In the Zhengdan 958 strain, the per gene also showed the highest expression in the flight muscle, followed by the head and antennae. The *clk* gene had the highest expression in the antennae, while the *cry* gene was most abundant in the antennae as well, followed by the head. The *cyc* gene peaked in the flight muscle, followed by the antennae and head. Additionally, the *per* gene’s expression was highest in the testis, followed by the head. The *clk* gene had the highest expression in the antennae, the cry gene was most expressed in the testis, and the cyc gene peaked in the foot.

**Figure 11:**
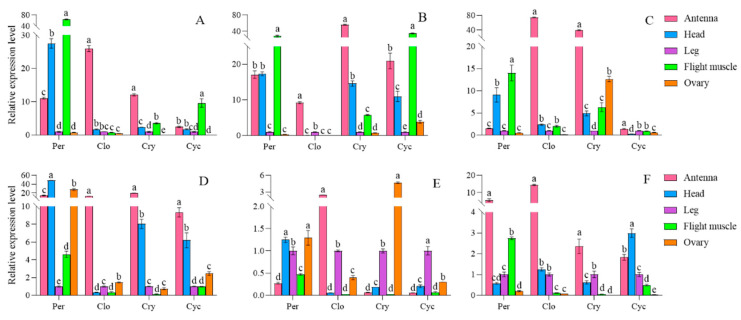
Relative expression levels of circadian clock genes in different tissues of *S. frugiperda* strains. (**A**–**F**). Relative expression levels of circadian clock genes (*clk*, *cry*, and *cyc* genes) in female and male adults of artificial diet, Zhengdan 958, and Zhenghuangnuo strains, respectively. The lowercase letters above the bars indicate the significance of differences in gene expression in different tissues.

In the Zhenghuangnuo strain, the per gene exhibited the highest expression in flight muscles, followed closely by the head. The *clk*, *cry*, and *cyc* genes showed their peak expression levels in the antennae. For males, the expression of *per*, *clk*, and *cry* genes was also highest in the antennae, while the *cyc* gene had the highest expression in the head, followed by the antennae. Across the three strains of *S. frugiperda*, the four *circadian clock* genes were predominantly expressed in the antennae and head. The expression patterns of these *circadian clock* genes in females were largely consistent across different strains. In males, the expression patterns of the artificial diet and Zhenghuangnuo strains were similar, differing from those observed in the Zhengdan 958 strain (Figure 11).

The expression levels of the four circadian clock genes varied across different strains and anatomical regions. In comparison to females from the artificial diet strain, *per* gene expression in the antennae, head, and feet of the Zhengdan 958 strain was higher and lower in the flight muscles and ovaries, respectively. For the Zhenghuangnuo strain, expression levels of the *per* gene decreased in the antennae, head, flight muscles, and ovaries but increased in the leg. The *clk* gene showed increased expression in the antennae and leg of the Zhengdan 958 strain, while it decreased in the head, flight muscle, and ovary. However, in the Zhenghuangnuo strain, *clk* gene expression was elevated in the antennae but reduced in the head, leg, flight muscle, and ovary. The expression levels of both *cry* and *cyc* genes in the Zhengdan 958 and Zhenghuangnuo strains decreased in the antennae, head, leg, and flight muscle, while they increased in the ovary.

**Figure 12:**
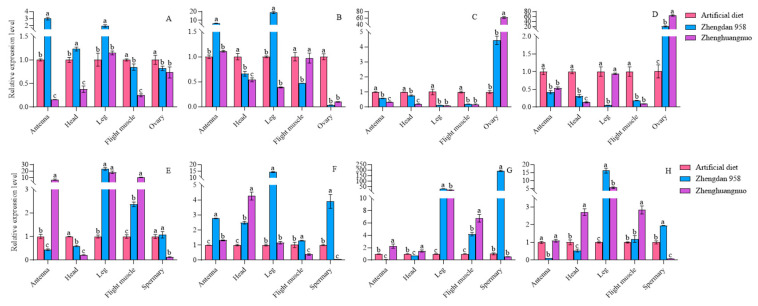
The relative expression of circadian clock genes in adults of the *S. frugiperda* strain. The expression levels of Per, Clk, Cry, and Cyc in female adults are represented by (**A**–**D**), while those in male adults are represented by (**E**–**H**), respectively. The lowercase letters above the bars indicate the significance of differences in gene expression in different strains.

When compared to males from the artificial diet strain, per gene expression in the antennae and head of the Zhengdan 958 strain decreased but increased in the feet, flight muscle, and testis. In the Zhenghuangnuo strain, expression levels were elevated in the antennae, leg, and flight muscles, while they decreased in the head and testis. *clk* gene expression rose across all tissues in the Zhengdan 958 strain. In the Zhenghuangnuo strain, it increased in the antennae, head, and leg, while it decreased in the flight muscle and testis. The levels of *cry* and *cyc* genes were reduced in the antennae and head of the Zhengdan 958 strain, yet they increased in the legs, flight muscles, and testes. In the Zhenghuangnuo strain, the expression was enhanced in the antennae, head, leg, and flight muscle, while it decreased in the testis (Figure 12).

## 4. Discussion

The majority of insects display distinct circadian rhythms in their emergence behavior. Nevertheless, there are significant variations in the eclosion patterns across different insect species. Typically, Lepidopteran insects emerge at night, but certain species have peak emergence times during the day. For example, *Lymantria dispar asiatica* Vnukovskij’s peak activity is between 7:00 and 10:00 [24], whereas that of *L. xylina* occurs between 10:00 and 12:00 and again from 14:00 to 16:00 [25]. The peak activity of *Cydia pomonella* Linnaeus is at 8:00–11:00 and 9:00–13:00 [26], while that of *Plutella xylostella* Linnaeus is at 6:00 and 10:00 [27]. *Iragoides fasciata* Moore, on the other hand, primarily emerges in the afternoon, specifically between 14:00 and 19:00 [28].

The eclosion rhythm of a single insect species can be influenced by various external factors, with different photoperiod treatments resulting in altered rhythms. For example, *Antheraea pernyi* Guérin-Meneville exhibited changes in its eclosion rhythm under varying photoperiods, with continuous light treatment showing no distinct periodicity [29]. *P. xylostella* subjected to photoperiod reversal lacked a regular emergence pattern [27], and *H*. *armigera* also displayed a reversed rhythm, primarily emerging during the day [30]. Additionally, plant secondary metabolites can impact insect rhythms; for instance, the methanol extract from indica rice affects serotonin levels in *S. frugiperda*, subsequently altering its behavioral and intestinal peristalsis rhythms [31].

Among the three strains, the emergence of female adults of *S. frugiperda* occurred earlier than that of males, aligning with observations in *S. litura* [32] and *Spodoptera exigua* Hübner [33]. However, in the cases of *L. dispar asiatica*, *L. xylina*, *C. pomonella*, *P. xylostella*, and *I. fasciata*, males preceded females in emergence [20,21,22,23,24,25,26,27,28]. This suggests that there were notable variations in the eclosion patterns among different Lepidopteran species.

The behavioral rhythms across the three strains varied, but resting was the predominant behavior observed. During the light period, all strains primarily rested with minimal activity, similar to observations in *Spodoptera litura* Fabricius [32] and *Athetis dissimilis* Hampson [34]. Although behavioral rhythms differ among insects, time allocation is primarily aimed at reducing energy expenditure [35].

*S. frugiperda* feeding on different corn cultivars over multiple generations resulted in variations in the ovarian development of newly emerged females. Those fed on the artificial diet exhibited a greater ovarian weight and length compared to those fed two types of corn leaves. *S. frugiperda* adults that consumed Zhenghuangnuo leaves had a significantly higher fat body weight and content than those fed on Zhengdan958 and the artificial diet. This suggests that the higher sugar content in Zhenghuangnuo leaves provides more nutrients conducive to fat accumulation during the larval stage. The artificial diet, rich in various nutrients and vitamins, supports ovarian development, leading to a superior ovary weight and length compared to the corn leaf strains. *S. frugiperda* adults exhibited different behavioral rhythms when feeding on various hosts, particularly in mating behavior, as noted in studies of *S. litura* [32] and *Chilo suppressalis* Walker [36,37]. Pashley identified differences in the mating rhythms between maize-type and rice-type *S. frugiperda*, which showed a preference for mating with their respective types [38]. These findings emphasize that the host plants on which insects feed can significantly influence their behavioral rhythms [33]. Recognizing these differences in the behavioral rhythms of pests on various host plants is essential for effective forecasting, prevention, control, and sexual trapping.

Xie discovered that the *HoPer* gene in *Holotrichia oblita* Faldermann exhibited high expression levels in the head and antennae, with female adults showing the highest expression in the antennae, while male adults had the highest expression in the head [39]. In *Bombyx mori* Linnaeus, the *cry* gene was predominantly expressed in the head antennae and flight muscles [40]. Ji investigated the expression of four circadian clock genes in *Mythimna separata* Walker, revealing that these genes were primarily expressed in the antennae and head, with varying expression patterns across different tissues [41]. The findings of this study indicated distinct expression levels of the four *circadian clock* genes in various strains of *S. frugiperda* adults across different tissues, predominantly in the antennae and head, aligning with previous results. Unlike Ji’s study, which showed similar expression patterns for the same genes in male and female adults of *M. separata*, this study found variations in expression patterns between genders, consistent with Xie’s findings. In *H. armigera*, the *cry1* gene was found to be highly expressed in the abdomen and less so in the antennae, while the *cry2* gene showed high expression in the thorax and low expression in the head [42]. In *C. suppressalis*, the *cry1* gene had high expression in the antennae, and the *cry2* gene was significantly expressed in the abdomen, followed by the head, with lower levels in the leg [43]. The silkworm exhibited high expression of the *cry1* gene in its head and antennae, while the *cry2* gene was more highly expressed in the head and flight muscle [40], indicating that the expression patterns of *circadian clock* genes vary across species.

Sweet and normal maize were used for the continuous rearing of *S. frugiperda* over nine generations. The expression levels of circadian clock genes in the two corn cultivars significantly differed from those in the artificial diet strain. The four circadian clock genes exhibited a decreasing trend in expression levels within the tissues of the two corn strains compared to the artificial diet strain, whereas the expression levels in male insects of the two strains showed an increasing trend. The expression patterns of the *cry* and *cyc* genes in the two maize strains were similar to those of the artificial diet strain. After multiple generations of continuous feeding, the expression differences in biological *clock* genes between the two maize strains and the artificial diet strain considerably increased compared to the first generation, indicating that prolonged feeding on different hosts amplifies the expression differences in insect biological clock genes. Dres and Mallet found that the same herbivorous insect can adapt to various host plants, with differing internal physiology, external morphology, and behavior among host strains potentially leading to reproductive isolation and the formation of distinct host strains within the same region [44]. Feeding on diverse hosts can also result in changes to insect-reproduction-related genes. Yao identified variations in the expression of the vitellogenin gene in *S. frugiperda* when feeding on different hosts [45], and similar differences were noted in *S. litura* [46]. This suggests that herbivorous insects feeding on various host plants can influence the expression of numerous genes, thereby impacting their behavior, reproduction, and other life processes. The results of this study demonstrate that *S. frugiperda* feeding on different host plants leads to variations in various behavioral rhythms. Further research has indicated that there are differences in the expression of circadian clock genes among adults of *S. frugiperda* from different host strains. This necessitates an additional investigation into the mechanisms behind these differences and highlights the importance of considering the influence of different hosts when predicting and implementing comprehensive pest control measures.

## 5. Conclusions

Interest in trap cropping, a traditional pest management tool, has increased significantly in recent years [47]. Attracting the major invasive pests- *S. frugiperda* by growing sweet and grain corn at the same time is a novel idea. In the Huang–Huai–Hai region of China, grain corn is mainly grown, and sweet and sticky corn can be used as a lure plant. Our results demonstrate that this dual-cropping approach might effectively lure *S. frugiperda* away from the main maize, thereby reducing pest pressure and the need for chemical pesticides. This method not only preserves the environment but also supports sustainable agriculture [48]. Future research should focus on optimizing trap crop placement, variety selection, and timing to maximize its effectiveness. Overall, sweet corn as a trap crop presents a promising alternative to conventional pest management strategies. The initial generation of *S. frugiperda* was cultivated using leaves from normal corn and sweet waxy corn. Compared to the strain fed an artificial diet, the behavioral rhythms of the three strains differed; however, they all exhibited activity during the resting dark period within the light phase. The RT-qPCR results are consistent with the behavioral rhythm findings, revealing variations in circadian clock gene expression among the three strains. After nine generations of continuous feeding on different maize leaf cultivars, behavioral rhythm differences persisted among the strains, though the time allocation for each behavior remained consistent, with resting behavior occupying the majority of the time. RT-qPCR analysis indicated that the expression levels of four circadian clock genes varied across different tissues in adult *S. frugiperda*, with the highest expression primarily found in the antennae and head. Significant differences in circadian clock gene expression were observed between the two maize cultivar strains and the artificial diet strain. In female insects from the two maize strains, the expression of the four circadian clock genes showed a decline compared to that in the artificial diet strain, while male insects from the two maize strains exhibited increased expression. The expression patterns of the *cry* and *cyc* genes in the two maize strains were similar to those in the artificial diet strain.

After the establishment of FAW, it will feed on corn for a long time, and using its rhythm difference will provide a scientific basis for field pest control. The results of this study emphasize the importance of understanding the feeding rhythms of the FAW in corn fields. By recognizing and exploiting these rhythms, farmers and pest control experts can develop more effective strategies to manage and mitigate the damage caused by this pest. Additionally, the findings contribute to the broader field of entomology by adding to our understanding of insect behavior and its ecological implications. Future research could further explore the potential of using rhythm differences in combination with other biological, cultural, or chemical control methods to enhance the overall effectiveness of pest management programs. Our findings suggest that the dietary environment, particularly the type of maize leaf consumed, can significantly influence the circadian clock gene expression and behavioral rhythms of *S. frugiperda*. The persistence of behavioral rhythm differences across generations highlights the potential for adaptive changes in the insects’ circadian systems in response to dietary cues. The tissue-specific expression patterns of circadian clock genes indicate that these genes may play different roles in different parts of the insect body, with the antennae and head potentially being key regions for circadian regulation. The observed sex-specific differences in circadian clock gene expression between the maize strains and the artificial diet strain suggest that dietary effects on circadian rhythms may also be sex-dependent. Overall, our study provides insights into the interplay between the dietary environment and circadian rhythms in insects, which may have implications for understanding the ecological and evolutionary consequences of dietary specialization in insects.

Our findings underscore the complexity of circadian regulation in insects and highlight the need for further research to fully elucidate the mechanisms underlying these interactions. Additionally, the observed sex-specific differences in circadian clock gene expression suggest that gender should be considered as a factor in studies examining the effects of the diet on circadian rhythms.

## Figures and Tables

**Table 1:** Information on circadian clock gene primers.

Primers	Primer Sequences (5′-3′)	Primer Usage
Per-F	ACCACTACAACTGTCGCATCG	RT-qPCR
Per-R	CACCGCCGGCATAGTGTG	
Clk-F	GAGTGCCACCCATTGATCCT	
Clk-R	CATCATCAGCGCGTAATCTGG	
Cry-F	CGCAATGCTTGGTGGTAGC	
Cry-R	CCAACCAGCTTTGTACCTGC	
Cyc-F	GGCTCATCGAGTCTCAGGTC	
Cyc-R	TGGCTTTTAGTGGCCAGTTCT	
Actin-F	TACTCCTAAGCCTGTTGATG	references
Actin-R	TTATGTCATGGTGCCGAAT	

**Table 2:** Effects of different maize cultivars on ovarian development of *S. frugiperda*.

Treatment	Ovary Weight (mg)	Fat Body Weight (mg)	Fat Body Content %	Ovarian Tube Length (mm)
Zhengdan 958	13.80 ± 0.60 Bb	12.20 ± 1.90 Aa	35.22 ± 3.51 ABab	39.95 ± 1.13 Aa
Zhenghuangnuo	22.10 ± 2.70 Aa	13.80 ± 1.70 Aa	43.36 ± 3.52 Aa	41.98 ± 0.75 Aa
Artificial diet	17.40 ± 2.80 Bb	6.70 ± 1.20 Bb	31.54 ± 3.87 Bb	35.66 ± 1.16 Bb

The data in the table are mean ± SE. Different capital and lowercase letters in the same row indicate extremely significant (*p* < 0.01) and significant (*p* < 0.05) differences among different *S. frugiperda* strains in the same treatment, respectively (Duncan’s multiple comparisons).

## Data Availability

The original contributions presented in this study are included in the article. Further inquiries can be directed to the corresponding authors.
